# Draft Genome Sequence of Anoxybacillus Strain BCO1, Isolated from a Thermophilic Microbial Mat Colonizing the Outflow of a Bore Well of the Great Artesian Basin of Australia

**DOI:** 10.1128/genomeA.01547-14

**Published:** 2015-02-12

**Authors:** Bharat K. C. Patel

**Affiliations:** Microbial Gene Research and Resources Facility, School of Natural Sciences, Griffith University, Brisbane, Queensland, Australia

## Abstract

Anoxybacillus strain BCO1, isolated from a thermophilic (50°C) microbial mat colonizing an outflow of a Great Artesian bore well of Australia, possessed a genome of ~2.8 Mb, with a G+C content of 41.7 mol%, and encoded 3,205 genes.

## GENOME ANNOUNCEMENT

The Great Artesian Basin (GAB) of Australia is the largest fresh and deepest subsurface geothermal water reservoir in the world. The water is used for agriculture and exploited for oil and gas. The aquifer is heated indirectly by the earth’s magmatic core up to boiling, and the water chemistry is influenced by the surrounding geologic formation (1), and this variation in temperature and chemistry makes the GAB an ideal ecosystem for investigations of thermophilic *Bacteria* and *Archaea* (2, 3).

Anoxybacillus strain BCO1 was isolated from a sample collected from a 50°C microbial mat colonizing a bore well outflow channel (2, 3). The strain was cultured in Medium D (4) under optimal growth conditions (50°C and pH 7.5) to mid-late log growth phase, the cells were centrifuged, and the DNA from the cell pellet was purified (5, 6). The genomic DNA of strain BCO1 consisting of 632,994 reads totaling 112 Mbp (112,602,909 bp) was generated using an Ion Torrent PGM sequencer and a 318 chip at the Australian Genome Research Facility (AGRF) core facility. High-quality filtered data was assembled using GS De Novo Assembler version 2.8; the generated genome of ~2.8 Mbp (34× coverage) consisting of 102 contigs with an average G+C content of 41.7 mol% was analyzed using RAST (7); gene annotation was done using Prokka version 1.10 (8); average nucleotide analysis was done with JSpecies (9); and e-DNA-DNA hybridization (e-DDH) was done using the Genome-to-Genome Distance Calculator (GGDC; http://ggdc.dsmz.de) (10).

RAST analysis predicted that the genome sequence comprised 3,205 putative protein-encoding genes, 47 tRNA genes, and 9 rRNA genes (5S rRNA, 16S rRNA, 23S rRNA). Of the 3,205 predicted proteins, 2,539 could be assigned probable biological functions, and the remaining 666 could not be assigned functions or were hypothetical proteins. Genes involved in the anaerobic metabolism of carbohydrates with putative proteins for the production of mixed acid and lactic acid as well as an electron transport chain involved in aerobic growth were identified, providing evidence of the strain’s heterotrophic and facultative anaerobic nature.

Strain BCO1 clustered with A. flavithermus strain 25, Anoxybacillus sp. strains SK3-4 and DT3, and A. kamachatkensis strain G10 (ANIb value, >94%). e-DDH between strain BCO1 and A. flavithermus strain 25 and Anoxybacillus sp. strain SK3-4 was 75%, whereas for Anoxybacillus sp. strain DT3 and A. kamachatkensis strain G10 the vales were 46% and 43%, respectively. However, interestingly, genes that encode the respiratory nitrate reductase complex are absent in strain BCO1, which is in common with A. flavithermus strain 25 and Anoxybacillus sp. strains SK3-4 and DT3 but different from A. kamachatkensis strain G10, which is reported to possess these genes (11). A more detailed comparative analysis of strain BCO1 with the related four strains will provide further insights into its adaptation and life in the thermophilic microbial mat community of a nonvolcanic thermal ecosystem.

### Nucleotide sequence accession numbers.

The Anoxybacillus isolate BCO1 whole-genome shotgun project has been deposited at DDBJ/EMBL/GenBank under the accession number JRLC00000000. The version described in this paper is version JRLC01000000.
